# Design, Synthesis, and CETP Inhibitory Evaluation of Novel P-methoxybenzene Sulfonamides Supported by Molecular Modeling

**DOI:** 10.5812/ijpr-170876

**Published:** 2026-10-17

**Authors:** Reema Abu Khalaf, Maha Awad, Mohammad Awad, Dima Sabbah, Ghadeer Albadawi

**Affiliations:** 1 Faculty of Pharmacy, Al-Zaytoonah University of Jordan Amman Jordan

**Keywords:** Cholesteryl Ester Transfer Protein, Hyperlipidemia, Induced-fit Docking, Pharmacophore Fingerprint, Sulfonamide Inhibitors

## Abstract

**Background:**

Hyperlipidemia is a global health concern characterized by elevated blood lipid concentrations, including cholesterol and triglycerides. Cholesteryl ester transfer protein (CETP) plays a crucial role in transferring cholesteryl esters and phospholipids from antiatherogenic high-density lipoproteins to atherogenic low-density and very-low-density lipoproteins.

**Objectives:**

This study aimed to synthesize and evaluate 20 p-methoxy-substituted benzene sulfonamides (6a-6j and 7a-7j) as potential CETP inhibitors.

**Methods:**

The compounds were synthesized and structurally characterized using infrared (IR) spectroscopy, carbon-13 nuclear magnetic resonance (^13^C-NMR) spectroscopy, proton nuclear magnetic resonance (^1^H-NMR) spectroscopy, and high-resolution mass spectrometry, followed by evaluation of CETP inhibitory activity. Molecular modeling studies were also performed.

**Results:**

The biological assay showed that inhibitors 7f-7j exhibited the highest inhibitory activity, achieving 100% inhibition at 10 µM; inhibitor 7j had an IC_50_ of 0.55 µM. Compounds 6a-6j and 7a-7e showed CETP inhibition of up to 95%. The presence of an additional ring markedly enhanced activity, likely due to the hydrophobic nature of CETP. The docking results supported this finding, indicating that ligand/4EWS complex formation was driven by hydrophobic interactions and hydrogen-bond formation with the amino acid residues H232 and R201. Higher binding scores were observed for series 7a-7j than for series 6a-6j, highlighting the importance of introducing an additional ring to enhance CETP inhibition. Furthermore, pharmacophore mapping confirmed that these analogues matched the fingerprint of active CETP inhibitors.

**Conclusions:**

A series of novel p-methoxybenzene sulfonamides was synthesized and evaluated as CETP inhibitors.

## 1. Background

Hyperlipidemia is a global disease characterized by elevated blood lipid concentrations, including cholesterol and triglycerides (1, 2). It is characterized by increased low-density lipoprotein (LDL) and/or very-low-density lipoprotein (VLDL) levels and decreased high-density lipoprotein (HDL) levels. Although lipids are essential for numerous bodily functions and organ structures, elevated lipid levels are harmful and are associated with several adverse consequences. Furthermore, the lipid profile significantly influences the progression of dyslipidemia in affected individuals (3).

Cardiovascular disease is the leading cause of death among adults in the United States. The incidence of cardiovascular disease in people with hyperlipidemia is almost twice that in individuals with healthy cholesterol levels (2-4). Atherosclerosis, caused by the accumulation of fatty plaques in the arteries, contributes to mortality worldwide and to conditions such as myocardial infarction, coronary heart disease, and stroke (5-7). Elevated LDL cholesterol and reduced HDL cholesterol levels are primary contributing factors to coronary heart disease incidence (8). These findings have led to the development of LDL cholesterol-lowering medications to prevent and treat atherosclerosis, with varying degrees of effectiveness (9). HDL cholesterol is inversely correlated with the risk of coronary artery disease and is an important predictor of cardiovascular disorders (10-12). The antiatherogenic effects of HDL are attributed to its roles in reverse cholesterol transport and the stimulation of cholesterol efflux, as well as its anti-inflammatory, anticoagulant, and antioxidant effects (13, 14).

Lowering atherogenic LDL cholesterol through lifestyle changes and pharmacological therapy, mainly statins, has considerably reduced heart disease (15, 16). Accordingly, reducing LDL cholesterol and increasing HDL cholesterol are treatment objectives for decreasing the prevalence of atherosclerosis associated with cardiovascular disease (17).

Cholesteryl ester transfer protein is a hydrophobic glycoprotein consisting of 476 residues. It is released from the liver into the blood and mediates the transfer of cholesteryl esters and phospholipids from antiatherogenic HDL to atherogenic LDL and VLDL. This process increases LDL and VLDL cholesterol levels while decreasing HDL cholesterol levels (18, 19). Consequently, cholesteryl ester transfer protein (CETP) inhibition has been proposed as a therapeutic target for treating dyslipidemia and reducing cardiovascular complications (20, 21). Several CETP inhibitors (Figure 1), including torcetrapib (22), anacetrapib (23), evacetrapib (22, 24), obicetrapib (25), and dalcetrapib (26), have been investigated. However, clinical studies of these inhibitors have been discontinued early because of increased mortality, cardiac complications, hypokalemia, and hypertension (27, 28).

**Figure 1.**
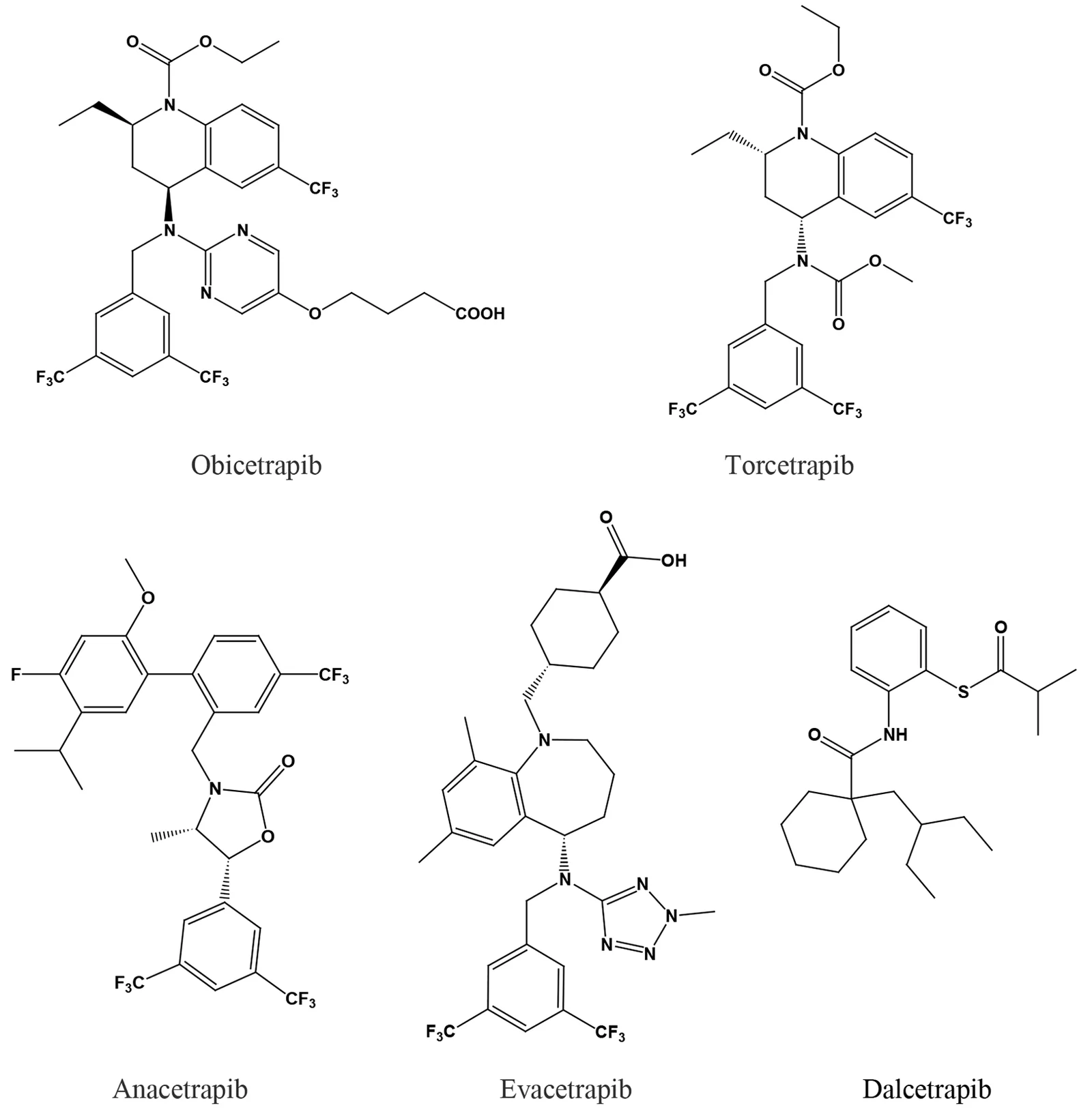
Structures of selected cholesteryl ester transfer protein (CETP) inhibitors.

Despite these challenges, CETP inhibition remains a promising target for managing hyperlipidemia and limiting its associated complications (10, 18, 29, 30). Our research group has previously designed, synthesized, and evaluated several compound series with promising CETP inhibitory activity, including benzylidene-aminomethanones (31), benzylamino-methanones (32), N-(4-benzyloxyphenyl)-4-methylbenzenesulfonamides, N-(4-benzylaminophenyl)-toluene-4-sulfonic acid esters (33), chlorobenzyl benzamides (34), substituted benzyl benzamides (35, 36), and fluorinated benzamides (37, 38).

Our team recently identified compounds A and B (Figure 2) as potential CETP inhibitors. These compounds demonstrated 100% CETP inhibition at 10 µM (39, 40).

**Figure 2.**
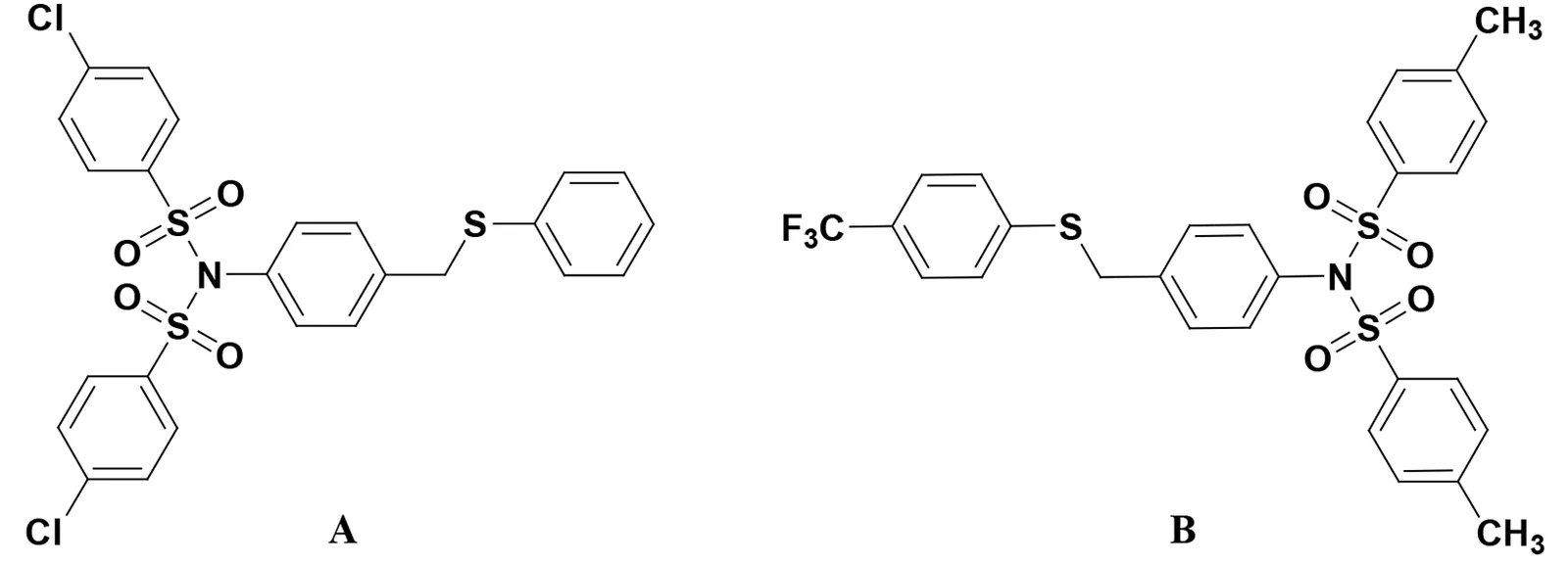
Cholesteryl ester transfer protein (CETP) inhibitors A and B (39, 40).

## 2. Objectives

This study aimed to investigate the effects of p-methoxy substitution on the thiophenyl ring on CETP inhibition and to further elucidate the structure–activity relationship of this series. Biological screening of the target compounds was also planned to identify new CETP inhibitory hits. A hit was defined as a compound with confirmed, reproducible activity in a biological assay, supported by dose–response data, and sufficient chemical integrity and tractability to justify progression toward lead optimization.

## 3. Methods

### 3.1. Materials and Analytical Methods

All solvents and chemicals were of analytical grade and were purchased from Acros, Fisher Scientific, Fluka, EMSURE, Sigma-Aldrich, Tedia, and Tokyo Chemical Industry through a local agent. A CETP inhibitor fluorometric screening kit was obtained from MyBioSource. Thin-layer chromatography sheets coated with silica gel 60 (0.2 mm thickness) were obtained from MACHEREY-NAGEL.

Proton (^1^H-NMR) and carbon-13 (^13^C-NMR) nuclear magnetic resonance spectra were recorded on a Bruker 500 MHz AVANCE III spectrometer. High-resolution mass spectrometry (HR-MS) was performed using a Bruker Impact II Q-TOF instrument. Infrared (IR) spectra were recorded using a Shimadzu Fourier-transform infrared (FT-IR) spectrophotometer. Activity was assessed using a BioTek FLx800 microplate fluorometer. Melting points were measured using a Griffin melting-point apparatus.

### 3.2. Chemical Preparation and Spectroscopic Characterization of Intermediate 4

4-Nitrobenzyl-4-methoxyphenyl sulfane (3) was obtained by reacting 4-nitrobenzyl bromide (1) with 4-methoxybenzenethiol (2). It was then reduced with sodium borohydride (NaBH_4_) to obtain 4-(((p-methoxyphenyl)thio)methyl)aniline (4) using the synthetic method described in our previous work (39, 40).

Intermediate 4 was obtained as a white powder (6.7 g, 59.0%); mp = 94 - 95 °C; Rƒ = 0.46 (DCM); ^1^H-NMR (500 MHz, DMSO-d_6_): δ 3.73 (s, 3H, OCH_3_), 3.94 (s, 2H, CH_2_), 4.99 (s, 2H, NH_2_), 6.46 (d, J = 10.0 Hz, 2H, Ar-H), 6.89 (dd, J = 12.0, 11.0 Hz, 4H, Ar-H), 7.26 ppm (d, J = 10.5 Hz, 2H, Ar-H); ^13^C-NMR (125 MHz, DMSO-d_6_): δ 39.4 (CH_2_), 55.6 (OCH_3_), 114.2, 115.0, 125.0, 127.0, 130.0, 133.0, 148.0, 159.0 ppm; FT-IR (KBr): 3387, 3321, 2962, 2920, 2837, 1625, 1597, 1496, 1437, 1288, 1176, 1095 cm^-1^. HR-MS (ESI, positive mode) m/z [M+H]^+^ 246.0581 (C_14_H_15_NOS + H) requires 246.0900.

### 3.3. Chemical Preparation and Spectroscopic Characterization of the Target Compounds

4-(((p-Methoxyphenyl)thio)methyl)aniline (2.0 mmol, 0.50 g) was dissolved in dichloromethane (DCM, 100 mL), followed by the addition of one of the sulfonyl chlorides (5a: 0.42 mL, 3.27 mmol; 5b: 0.47 mL, 3.28 mmol; 5c: 0.47 mL, 3.28 mmol; 5d: 0.42 mL, 2.93 mmol; 5e: 0.45 mL, 3.17 mmol; 5f: 0.47 mL, 3.31 mmol; 5g: 0.69 g, 3.25 mmol; 5h: 0.43 mL, 3.27 mmol; 5i: 0.43 mL, 3.27 mmol; or 5j: 0.43 mL, 3.27 mmol) and TEA (7.61 mmol, 1.06 mL). The reaction mixture was stirred for 1 - 3 days at room temperature. The solvent was then evaporated at 50 - 70 °C. The target compounds were purified by column chromatography using dichloromethane:cyclohexane (8:2) as the mobile phase (39, 40).

3.3.1. N-(4-(((p-Methoxyphenyl)thio)methyl)phenyl)benzenesulfonamide (6a) and N-(4-(((p-methoxyphenyl)thio)methyl)phenyl)-N-(phenylsulfonyl)benzenesulfonamide (7a)

6a was obtained as a white powder (0.18 g, 15.3%); mp = 108 - 109 °C; Rƒ = 0.51 (DCM); ^1^H-NMR (500 MHz, DMSO-d_6_): δ 3.72 (s, 3H, OCH_3_), 3.96 (s, 2H, CH_2_), 6.82 (d, J = 11.0 Hz, 2H, Ar-H), 6.98 (d, J = 10.5 Hz, 2H, Ar-H), 7.08 (d, J = 10.5 Hz, 2H, Ar-H), 7.18 (d, J = 11.0 Hz, 2H, Ar-H), 7.53 (t, J = 10.0 Hz, 2H, Ar-H), 7.60 (t, J = 8.5 Hz, 1H, Ar-H), 7.73 (d, J = 9.0 Hz, 2H, Ar-H), 10.23 (s, 1H, N-H); ^13^C-NMR (125 MHz, DMSO-d_6_): δ 39.0 (CH_2_), 55.6 (OCH_3_), 115.0, 121.0, 126.0, 127.1, 129.7, 130.0, 133.1, 133.3, 134.3, 136.8, 139.9, 159.0 ppm; FT-IR (KBr): 3317, 3042, 2964, 1614, 1593, 1512, 1492, 1446, 1334, 1163 cm^-1^. HR-MS (ESI, positive mode) m/z [M+Na]^+^ 408.0672 (C_20_H_19_NO_3_S_2_ + Na) requires 408.0806.

7a was obtained as a white powder (0.2 g, 12.5%); mp = 98 - 99 °C; Rƒ = 0.5 (DCM); ^1^H-NMR (500 MHz, DMSO-d_6_): δ 3.72 (s, 3H, OCH_3_), 4.12 (s, 2H, CH_2_), 6.89 (dd, J = 6.0, 5.0 Hz, 4H, Ar-H), 7.26 (d, J = 10.5 Hz, 4H, Ar-H), 7.68 (t, J = 9.5 Hz, 4H, Ar-H), 7.78 - 7.85 ppm (m, 6H, Ar-H); ^13^C-NMR (125 MHz, DMSO-d_6_): δ 39.0 (CH_2_), 55.7 (OCH_3_), 115.1, 125.4, 128.4, 130.0, 130.3, 131.6, 132.3, 133.6, 135.2, 138.9, 141.4 ppm; FT-IR (KBr): 3062, 2962, 2841, 1587, 1492, 1444, 1381, 1282, 1166 cm^-1^. HR-MS (ESI, positive mode) m/z [M+NH_4_]^+^ 543.1051 (C_26_H_23_NO_5_S_3_ + NH_4_) requires 543.0738.

3.3.2. N-(4-(((p-Methoxyphenyl)thio)methyl)phenyl)-o-methylbenzenesulfonamide (6b) and N-(4-(((p-methoxyphenyl)thio)methyl)phenyl)-o-methyl-N-(o-tolylsulfonyl)benzenesulfonamide (7b)

6b was obtained as a white powder (0.20 g, 21%); mp = 95 - 96 °C; Rƒ = 0.42 (DCM); ^1^H-NMR (500 MHz, DMSO-d_6_): δ 2.57 (s, 3H, CH_3_), 3.72 (s, 3H, OCH_3_), 3.95 (s, 2H, CH_2_), 6.82 (d, J = 11.0 Hz, 2H, Ar-H), 6.96 (d, J = 10.5 Hz, 2H, Ar-H), 7.06 (d, J = 10.5 Hz, 2H, Ar-H), 7.17 (d, J = 11.0 Hz, 2H, Ar-H), 7.34 (t, J = 10.0 Hz, 2H, Ar-H), 7.48 (t, J = 8.5 Hz, 1H, Ar-H), 7.85 (d, J = 9.5 Hz, 1H, Ar-H), 10.36 (s, 1H, N-H); ^13^C-NMR (125 MHz, DMSO-d_6_): δ 20.2 (CH_3_), 38.9 (CH_2_), 55.6 (OCH_3_), 115.0, 119.4, 126.0, 126.7, 129.8, 130.0, 133.0, 133.4, 133.7, 136.8, 137.2, 138.0, 159.0 ppm; FT-IR (KBr): 3317, 3048, 2966, 2845, 1598, 1504, 1396, 1338, 1232, 1153 cm^-1^. HR-MS (ESI, positive mode) m/z [M+NH_4_]^+^ 417.1273 (C_21_H_21_NO_3_S_2_ + NH_4_) requires 417.0963.

7b was obtained as a white powder (0.51 g, 31%); mp = 90 - 91 °C; Rƒ = 0.54 (DCM); ^1^H-NMR (500 MHz, DMSO-d_6_): δ 2.24 (s, 6H, 2×CH_3_), 3.75 (s, 3H, OCH_3_), 4.10 (s, 2H, CH_2_), 6.87 (d, J = 11.0 Hz, 2H, Ar-H), 6.97 (d, J = 12.0 Hz, 2H, Ar-H), 7.23 (d, J = 11.0 Hz, 4H, Ar-H), 7.40 - 7.46 (m, 4H, Ar-H), 7.66 (t, J = 9.5 Hz, 2H, Ar-H), 7.86 ppm (d, J = 10.0 Hz, 2H, Ar-H); ^13^C-NMR (125 MHz, DMSO-d_6_): δ 20.3 (2×CH_3_), 38.8 (CH_2_), 55.7 (OCH_3_), 115.1, 125.3, 127.2, 127.2, 130.2, 131.2, 131.5, 131.8, 133.2, 133.4, 135.1, 136.8, 138.7, 141.5, 159.2 ppm; FT-IR (KBr): 3167, 3078, 3030, 2933, 2835, 1591, 1492, 1431, 1348, 1242, 1161 cm^-1^. HR-MS (ESI, positive mode) m/z [M+NH_4_]^+^ 571.1364 (C_28_H_27_NO_5_S_3_ + NH_4_) requires 571.1051.

3.3.3. N-(4-(((p-Methoxyphenyl)thio)methyl)phenyl)-m-methylbenzenesulfonamide (6c) and N-(4-(((p-methoxyphenyl)thio)methyl)phenyl)-m-methyl-N-(m-tolylsulfonyl)benzenesulfonamide (7c)

6c was obtained as a white powder (0.16 g, 13.4%); mp = 94 - 95 °C; Rƒ = 0.42 (DCM); ^1^H-NMR (500 MHz, DMSO-d_6_): δ 2.33 (s, 3H, CH_3_), 3.72 (s, 3H, OCH_3_), 3.97 (s, 2H, CH_2_), 6.82 (d, J = 11.0 Hz, 2H, Ar-H), 6.97 (d, J = 9.0 Hz, 2H, Ar-H), 7.08 (d, J = 10.5 Hz, 2H, Ar-H), 7.19 (d, J = 11.0 Hz, 2H, Ar-H), 7.41 (d, J = 6.0 Hz, 2H, Ar-H), 7.50 - 7.52 (m, 1H, Ar-H), 7.60 (s, 1H, Ar-H), 10.19 (s, 1H, N-H); ^13^C-NMR (125 MHz, DMSO-d_6_): δ 21.3 (CH_3_), 39.0 (CH_2_), 55.6 (OCH_3_), 115.0, 120.4, 124.3, 126.0, 127.3, 129.5, 130.0, 133.0, 134.0, 134.1, 137.0, 139.4, 140.0 ppm; FT-IR (KBr): 3277, 3061, 2935, 2831, 1672, 1606, 1591, 1512, 1338, 1149 cm^-1^. HR-MS (ESI, positive mode) m/z [M+NH_4_]^+^ 417.1276 (C_21_H_21_NO_3_S_2_ + NH_4_) requires 417.0963.

7c was obtained as a white powder (0.73 g, 47%); mp = 106 - 107 °C; Rƒ = 0.5 (DCM); ^1^H-NMR (500 MHz, DMSO-d_6_): δ 2.40 (s, 6H, 2×CH_3_), 3.73 (s, 3H, OCH_3_), 4.13 (s, 2H, CH_2_), 6.88 (t, J = 11.0 Hz, 4H, Ar-H), 7.27 (dd, J = 11.0, 4.5 Hz, 4H, Ar-H), 7.52 - 7.57 (m, 6H, Ar-H), 7.64 ppm (s, 2H, Ar-H); ^13^C-NMR (125 MHz, DMSO-d_6_): δ 21.2 (2×CH_3_), 39.0 (CH_2_), 55.7 (OCH_3_), 115.1, 125.5, 126.0, 128.4, 130.0, 130.3, 131.6, 132.4, 133.4, 135.7, 139.0, 139.9, 141.3, 159.2 ppm; FT-IR (KBr): 3078, 3059, 2960, 2933, 2835, 1587, 1496, 1369, 1290, 1172, 1163 cm^-1^. HR-MS (ESI, positive mode) m/z [M+NH_4_]^+^ 571.1351 (C_28_H_27_NO_5_S_3_ + NH_4_) requires 571.1051.

3.3.4. N-(4-(((p-Methoxyphenyl)thio)methyl)phenyl)-p-methylbenzenesulfonamide (6d) and N-(4-(((p-methoxyphenyl)thio)methyl)phenyl)-p-methyl-N-tosylbenzenesulfonamide (7d)

6d was obtained as a white powder (0.25 g, 21%); mp = 125 - 126 °C; Rƒ = 0.42 (DCM); ^1^H-NMR (500 MHz, DMSO-d_6_): δ 2.33 (s, 3H, CH_3_), 3.72 (s, 3H, OCH_3_), 3.97 (s, 2H, CH_2_), 6.82 (d, J = 11.0 Hz, 2H, Ar-H), 6.97 (d, J = 10.5 Hz, 2H, Ar-H), 7.07 (d, J = 10.5 Hz, 2H, Ar-H), 7.19 (d, J = 11.0 Hz, 2H, Ar-H), 7.32 (d, J = 10.0 Hz, 2H, Ar-H), 7.60 (d, J = 10.5 Hz, 2H, Ar-H), 10.14 (s, 1H, N-H); ^13^C-NMR (125 MHz, DMSO-d_6_): δ 21.4 (2×CH_3_), 39.9 (CH_2_), 55.6 (OCH_3_), 115.0, 120.4, 126.0, 127.1, 130.0, 130.1, 133.1, 134.1, 137.0, 137.1, 143.6, 159.0 ppm; FT-IR (KBr): 3278, 3014, 2964, 2839, 1589, 1566, 1508, 1442, 1392, 1330, 1261, 1157 cm^-1^. HR-MS (ESI, negative mode) m/z [M-H]^-^ 398.0867 (C_21_H_21_NO_3_S_2_ - H) requires 398.0963.

7d was obtained as a white powder (0.31 g, 18%); mp = 143 - 144 °C; Rƒ = 0.54 (DCM); ^1^H-NMR (500 MHz, DMSO-d_6_): δ 2.49 (s, 6H, 2×CH_3_), 3.74 (s, 3H, OCH_3_), 4.12 (s, 2H, CH_2_), 6.88 (dd, J = 6.5, 4.0 Hz, 4H, Ar-H), 7.26 (dd, J = 7.0, 3.5 Hz, 4H, Ar-H), 7.46 (d, J = 10.0 Hz, 4H, Ar-H), 7.65 ppm (d, J = 10.5 Hz, 4H, Ar-H); ^13^C-NMR (125 MHz, DMSO-d^6^): δ 21.6 (2×CH_3_), 39.0 (CH_2_), 56.0 (OCH_3_), 115.1, 125.4, 128.5, 130.2, 130.4, 131.5, 132.5, 133.5, 136.2, 141.2, 146.0, 159.2 ppm; FT-IR (KBr): 3066, 2962, 2926, 2839, 1595, 1573, 1494, 1371, 1168, 1103 cm^-1^. HR-MS (ESI, positive mode) m/z [M+NH_4_]^+^ 571.1340 (C_28_H_27_NO_5_S_3_ + NH_4_) requires 571.1051.

3.3.5. o-Chloro-N-(4-(((p-methoxyphenyl)thio)methyl)phenyl)benzenesulfonamide (6e) and o-chloro-N-((o-chlorophenyl)sulfonyl)-N-(4-(((p-methoxyphenyl)thio)methyl)phenyl)benzene sulfonamide (7e)

6e was obtained as a white powder (0.46 g, 38%); mp = 112 - 113 °C; Rƒ = 0.6 (DCM); ^1^H-NMR (500 MHz, DMSO-d_6_): δ 3.72 (s, 3H, OCH_3_), 3.95 (s, 2H, CH_2_), 6.82 (d, J = 11.0 Hz, 2H, Ar-H), 6.98 (d, J = 10.5 Hz, 2H, Ar-H), 7.07 (d, J = 10.5 Hz, 2H, Ar-H), 7.16 (d, J = 11.0 Hz, 2H, Ar-H), 7.48 - 7.54 (m, 1H, Ar-H), 7.62 (d, J = 5.0 Hz, 2H, Ar-H), 8.01 (d, J = 10.0 Hz, 1H, Ar-H), 10.56 (s, 1H, N-H); ^13^C-NMR (125 MHz, DMSO-d_6_): δ 39.0 (CH_2_), 55.6 (OCH_3_), 115.0, 120.0, 126.0, 128.1, 130.0, 131.2, 132.1, 132.3, 133.1, 134.1, 135.1, 136.2, 137.0 ppm; FT-IR (KBr): 3277, 3089, 2962, 2920, 2846, 1589, 1510, 1492, 1400, 1338, 1282, 1163 cm^-1^. HR-MS (ESI, positive mode) m/z [M+NH_4_]^+^ 437.0729 (C_20_H_18_ClNO_3_S_2_ + NH_4_) requires 437.0417.

7e was obtained as a white powder (0.264 g, 15%); mp = 160 - 161 °C; Rƒ = 0.46 (DCM); ^1^H-NMR (500 MHz, DMSO-d_6_): δ 3.75 (s, 3H, OCH_3_), 4.08 (s, 2H, CH_2_), 6.87 (d, J = 11.0 Hz, 2H, Ar-H), 7.11 (d, J = 10.5 Hz, 2H, Ar-H), 7.22 (dd, J = 6.0, 5.0 Hz, 4H, Ar-H), 7.58 (t, J = 10.5 Hz, 2H, Ar-H), 7.72 (d, J = 10.0 Hz, 2H, Ar-H), 7.79 (t, J = 10.5 Hz, 2H, Ar-H), 7.98 ppm (dd, J = 10.5, 8.5 Hz, 2H, Ar-H); ^13^C-NMR (125 MHz, DMSO-d_6_): δ 39.0 (CH_2_), 55.7 (OCH_3_), 115.1, 125.3, 128.4, 130.1, 132.1, 132.5, 133.0, 133.5, 133.7, 135.2, 137.0, 159.2 ppm; FT-IR (KBr): 3088, 3024, 2964, 2902, 2837, 1579, 1492, 1444, 1369, 1288, 1172 cm^-1^. HR-MS (ESI, positive mode) m/z [M+NH_4_]^+^ 611.0202 (C_26_H_21_Cl_2_NO_5_S_3_ + NH_4_) requires 610.9956.

3.3.6. m-Chloro-N-(4-(((p-methoxyphenyl)thio)methyl)phenyl)benzenesulfonamide (6f) and m-chloro-N-((m-chlorophenyl)sulfonyl)-N-(4-(((p-methoxyphenyl)thio)methyl)phenyl)benzene sulfonamide (7f)

6f was obtained as a white powder (0.18 g, 26%); mp = 95 - 96 °C; Rƒ = 0.69 (DCM); ^1^H-NMR (500 MHz, DMSO-d_6_): δ 3.67 (s, 3H, OCH_3_), 3.93 (s, 2H, CH_2_), 6.77 (d, J = 8.5 Hz, 2H, Ar-H), 6.93 (d, J = 8.15 Hz, 2H, Ar-H), 7.05 (d, J = 8.15 Hz, 2H, Ar-H), 7.13 (d, J = 8.45 Hz, 2H, Ar-H), 7.53 (t, J = 7.85 Hz, 1H, Ar-H), 7.61 (d, J = 7.8 Hz, 1H, Ar-H), 7.65 (s, 1H, Ar-H), 7.67 (d, J = 8.2 Hz, 1H, Ar-H), 10.28 (s, 1H, N-H); ^13^C-NMR (125 MHz, DMSO-d_6_): δ 39.0 (CH_2_), 55.6 (OCH_3_), 114.2, 115.0, 121.0, 126.0, 126.6, 130.1, 132.0, 133.2, 133.4, 134.3, 135.0, 136.3, 141.7, 159.0 ppm; FT-IR (KBr): 3277, 3089, 2962, 2920, 2846, 1589, 1490, 1400, 1338, 1282, 1163 cm^-1^. HR-MS (ESI, negative mode) m/z [M-H]^-^ 418.0350 (C_20_H_18_ClNO_3_S_2_ - H) requires 418.0417.

7f was obtained as a white powder (0.62 g, 44%); mp = 110 - 111 °C; Rƒ = 0.5 (DCM); ^1^H-NMR (500 MHz, DMSO-d_6_): δ 3.31 (s, 3H, OCH_3_), 4.10 (s, 2H, CH_2_), 6.84 (d, J = 8.15 Hz, 2H, Ar-H), 6.93 (d, J = 8.15 Hz, 2H, Ar-H), 7.23 (dd, J = 11.9, 8.35 Hz, 4H, Ar-H), 7.71 (d, J = 14.9 Hz, 6H, Ar-H), 7.90 ppm (s, 2H, Ar-H); ^13^C-NMR (125 MHz, DMSO-d_6_): δ 39.0 (CH_2_), 55.7 (OCH_3_), 115.1, 125.4, 127.3, 128.0, 130.5, 131.6, 132.0, 132.2, 134.0, 134.6, 135.4, 140.2, 142.0, 159.2 ppm; FT-IR (KBr): 3093, 3066, 2958, 2937, 2833, 1591, 1573, 1492, 1392, 1290, 1168 cm^-1^. HR-MS (ESI, positive mode) m/z [M+NH_4_]^+^ 611.0219 (C_26_H_21_Cl_2_NO_5_S_3_ + NH_4_) requires 610.9959.

3.3.7. p-Chloro-N-(4-(((p-methoxyphenyl)thio)methyl)phenyl)benzenesulfonamide (6g) and p-chloro-N-((p-chlorophenyl)sulfonyl)-N-(4-(((p-methoxyphenyl)thio)methyl)phenyl)benzene sulfonamide (7g)

6g was obtained as a white powder (0.38 g, 32%); mp = 100 - 101 °C; Rƒ = 0.7 (DCM); ^1^H-NMR (500 MHz, DMSO-d_6_): δ 3.67 (s, 3H, OCH_3_), 3.89 (s, 2H, CH_2_), 6.77 (d, J = 8.5 Hz, 2H, Ar-H), 6.92 (d, J = 8.20 Hz, 2H, Ar-H), 7.04 (d, J = 8.20 Hz, 2H, Ar-H), 7.13 (d, J = 8.50 Hz, 2H, Ar-H), 7.56 (d, J = 8.35 Hz, 2H, Ar-H), 7.66 (d, J = 8.40 Hz, 2H, Ar-H), 10.23 ppm (s, 1H, N-H); ^13^C-NMR (125 MHz, DMSO-d_6_): δ 39.0 (CH_2_), 55.6 (OCH_3_), 115.0, 121.0, 126.0, 129.1, 129.9, 130.1, 132.6, 133.2, 134.7, 136.5, 138.2, 159.0 ppm; FT-IR (KBr): 3265, 3062, 2962, 2833, 1591, 1571, 1492, 1444, 1394, 1282, 1165 cm^-1^. HR-MS (ESI, negative mode) m/z [M-H]^-^ 418.0364 (C_20_H_18_ClNO_3_S_2_ - H) requires 418.0417.

7g was obtained as a white powder (0.78 g, 44%); mp = 155 - 156 °C; Rƒ = 0.68 (DCM); ^1^H-NMR (500 MHz, DMSO-d_6_): δ 3.84 (s, 3H, OCH_3_), 4.22 (s, 2H, CH_2_), 6.84 (d, J = 8.55 Hz, 2H, Ar-H), 6.90 (d, J = 8.05 Hz, 2H, Ar-H), 7.23 (t, J = 8.25 Hz, 4H, Ar-H), 7.75 ppm (dd, J = 11.95, 8.60 Hz, 8H, Ar-H); ^13^C-NMR (125 MHz, DMSO-d_6_): δ 39.0 (CH_2_), 55.7 (OCH_3_), 115.1, 125.4, 130.3, 130.4, 130.5, 131.5, 132.0, 133.6, 137.5, 140.3, 140.3, 141.7, 159.2 ppm; FT-IR (KBr): 3089, 2960, 2835, 1589, 1571, 1490, 1392, 1284, 1165 cm^-1^. HR-MS (ESI, positive mode) m/z [M+NH_4_]^+^ 611.0237 (C_26_H_21_Cl_2_NO_5_S_3_ + NH_4_) requires 610.9956.

3.3.8. o-Fluoro-N-(4-(((p-methoxyphenyl)thio)methyl)phenyl)benzenesulfonamide (6h) and o-fluoro-N-((o-fluorophenyl)sulfonyl)-N-(4-(((p-methoxyphenyl)thio)methyl)phenyl)benzene sulfonamide (7h)

6h was obtained as a white powder (0.46 g, 38%); mp = 119 - 120 °C; Rƒ = 0.5 (DCM); ^1^H-NMR (500 MHz, DMSO-d_6_): δ 3.70 (s, 3H, OCH_3_), 3.95 (s, 2H, CH_2_), 6.80 (d, J = 8.40 Hz, 2H, Ar-H), 6.98 (d, J = 8.15 Hz, 2H, Ar-H), 7.07 (d, J = 8.00 Hz, 2H, Ar-H), 7.16 (d, J = 8.35 Hz, 2H, Ar-H), 7.32 (t, J = 7.50 Hz, 1H, Ar-H), 7.38 (t, J = 8.80 Hz, 1H, Ar-H), 7.66 (d, J = 5.45 Hz, 1H, Ar-H), 7.77 (t, J = 7.35 Hz, 1H, Ar-H), 10.65 ppm (s, 1H, N-H); ^13^C-NMR (125 MHz, DMSO-d_6_): δ 39.0 (CH_2_), 55.6 (OCH_3_), 115.0, 117.6, 120.2, 125.4, 125.9, 127.4, 130.1, 130.9, 133.1, 134.4, 136.3, 157.6, 158.9, 159.6 ppm; FT-IR (KBr): 3259, 3093, 3053, 2933, 1639, 1587, 1571, 1473, 1344, 1284, 1244, 1168 cm^-1^. HR-MS (ESI, positive mode) m/z [M+H]^+^ 404.0793 (C_20_H_18_FNO_3_S_2_ + H) requires 404.0712.

7h was obtained as a white powder (0.85 g, 50%); mp = 130 - 131 °C; Rƒ = 0.56 (DCM); ^1^H-NMR (500 MHz, DMSO-d_6_): δ 3.73 (s, 3H, OCH_3_), 4.10 (s, 2H, CH_2_), 6.86 (d, J = 8.40 Hz, 2H, Ar-H), 7.07 (d, J = 7.85 Hz, 2H, Ar-H), 7.24 (d, J = 8.40 Hz, 2H, Ar-H), 7.26 (d, J = 7.90 Hz, 2H, Ar-H), 7.43 (t, J = 7.70 Hz, 2H, Ar-H), 7.49 (t, J = 9.15 Hz, 2H, Ar-H), 7.80 (t, J = 7.35 Hz, 2H, Ar-H), 7.87 ppm (d, J = 4.50 Hz, 2H, Ar-H); ^13^C-NMR (125 MHz, DMSO-d_6_): δ 39.0 (CH_2_), 55.7 (OCH_3_), 115.1, 118.0, 125.4, 125.7, 126.0, 130.2, 131.2, 131.7, 132.0, 133.6, 138.5, 141.7, 157.7, 159.2, 159.8 ppm; FT-IR (KBr): 3095, 3028, 2958, 2931, 2841, 1643, 1591, 1489, 1377, 1284, 1174 cm^-1^. HR-MS (ESI, positive mode) m/z [M+H]^+^ 562.0623 (C_26_H_21_F_2_NO_5_S_3_ + H) requires 562.0550.

3.3.9. m-Fluoro-N-(4-(((p-methoxyphenyl)thio)methyl)phenyl)benzenesulfonamide (6i) and m-fluoro-N-((m-fluorophenyl)sulfonyl)-N-(4-(((p-methoxyphenyl)thio)methyl)phenyl)benzene sulfonamide (7i)

6i was obtained as a white powder (0.57 g, 47.2%); mp = 112 - 113 °C; Rƒ = 0.5 (DCM); ^1^H-NMR (500 MHz, DMSO-d_6_): δ 3.71 (s, 3H, OCH_3_), 3.96 (s, 2H, CH_2_), 6.81 (d, J = 7.90 Hz, 2H, Ar-H), 6.97 (d, J = 7.55 Hz, 2H, Ar-H), 7.08 (d, J = 7.40 Hz, 2H, Ar-H), 7.17 (d, J = 7.90 Hz, 2H, Ar-H), 7.49 (d, J = 8.05 Hz, 2H, Ar-H), 7.56 (t, J = 7.75 Hz, 1H, Ar-H), 7.60 (t, J = 3.85 Hz, 1H, Ar-H), 10.65 ppm (s, 1H, N-H); ^13^C-NMR (125 MHz, DMSO-d_6_): δ 39.0 (CH_2_), 55.6 (OCH_3_), 115.0, 120.7, 121.0, 123.4, 125.8, 130.1, 132.1, 133.2, 134.7, 136.4, 141.9, 159.0, 161.1 ppm; FT-IR (KBr): 3286, 2962, 2931, 2839, 1589, 1566, 1490, 1442, 1334, 1282, 1263, 1168 cm^-1^. HR-MS (ESI, positive mode) m/z [M+H]^+^ 404.0785 (C_20_H_18_FNO_3_S_2_ + H) requires 404.0712.

7i was obtained as a white powder (0.73 g, 43%); mp = 144 - 145 °C; Rƒ = 0.46 (DCM); ^1^H-NMR (500 MHz, DMSO-d_6_): δ 3.72 (s, 3H, OCH_3_), 4.12 (s, 2H, CH_2_), 6.86 (d, J = 7.90 Hz, 2H, Ar-H), 6.97 (d, J = 7.60 Hz, 2H, Ar-H), 7.26 (dd, J = 8.00, 7.65 Hz, 4H, Ar-H), 7.58 (d, J = 7.75 Hz, 2H, Ar-H), 7.64 (d, J = 6.40 Hz, 2H, Ar-H), 7.74 ppm (t, J = 7.05 Hz, 4H, Ar-H); ^13^C-NMR (125 MHz, DMSO-d_6_): δ 39.0 (CH_2_), 55.6 (OCH_3_), 115.1, 115.4, 122.6, 125.0, 130.5, 131.5, 131.8, 132.6, 133.6, 140.4, 141.8, 159.2, 161.0, 163.0 ppm; FT-IR (KBr): 3062, 2960, 2841, 1593, 1487, 1429, 1390, 1296, 1157 cm^-1^. HR-MS (ESI, positive mode) m/z [M+H]^+^ 562.0623 (C_26_H_21_F_2_NO_5_S_3_ + H) requires 562.0550.

3.3.10. p-Fluoro-N-(4-(((p-methoxyphenyl)thio)methyl)phenyl)benzenesulfonamide (6j) and p-fluoro-N-((p-fluorophenyl)sulfonyl)-N-(4-(((p-methoxyphenyl)thio)methyl)phenyl)benzene sulfonamide (7j)

6j was obtained as a white powder (0.27 g, 34%); mp = 115 - 116 °C; Rƒ = 0.6 (DCM); ^1^H-NMR (500 MHz, DMSO-d_6_): δ 3.73 (s, 3H, OCH_3_), 4.11 (s, 2H, CH_2_), 6.87 (d, J = 8.55 Hz, 2H, Ar-H), 7.10 (d, J = 8.15 Hz, 2H, Ar-H), 7.28 (dd, J = 8.60, 7.05 Hz, 4H, Ar-H), 8.02 (t, J = 8.65 Hz, 4H, Ar-H), 8.13 (t, J = 4.85 Hz, 2H, Ar-H), 8.18 ppm (t, J = 3.45 Hz, 2H, Ar-H); ^13^C-NMR (125 MHz, DMSO-d_6_): δ 39.2 (CH_2_), 55.7 (OCH_3_), 111.1, 115.1, 125.6, 130.5, 132.1, 133.6, 134.6, 136.1, 136.7, 138.9, 142.3, 159.2 ppm; FT-IR (KBr): 3253, 3103, 3028, 2926, 1604, 1568, 1448, 1350, 1278, 1163 cm^-1^. HR-MS (ESI, positive mode) m/z [M+H]^+^ 404.0785 (C_20_H_18_FNO_3_S_2_ + H) requires 404.0712.

7j was obtained as a white powder (0.81 g, 48%); mp = 140 - 141 °C; Rƒ = 0.56 (DCM); ^1^H-NMR (500 MHz, DMSO-d_6_): δ 3.72 (s, 3H, OCH_3_), 4.12 (s, 2H, CH_2_), 6.90 (dd, J = 8.35, 3.85 Hz, 4H, Ar-H), 7.26 (t, J = 5.20 Hz, 4H, Ar-H), 7.51 (t, J = 8.50 Hz, 4H, Ar-H), 7.86 ppm (t, J = 4.95 Hz, 4H, Ar-H); ^13^C-NMR (125 MHz, DMSO-d_6_): δ 38.9 (CH_2_), 55.7 (OCH_3_), 115.1, 117.3, 125.4, 130.5, 131.5, 131.7, 133.5, 135.1, 141.5, 159.2, 164.8, 166.9 ppm; FT-IR (KBr): 3105, 3064, 2960, 2841, 1591, 1487, 1431, 1388, 1294, 1159 cm^-1^. HR-MS (ESI, positive mode) m/z [M+H]^+^ 562.0623 (C_26_H_21_F_2_NO_5_S_3_ + H) requires 562.0550.

### 3.4. CETP Inhibitory Activity Assay

CETP inhibitory activity was evaluated using a MyBioSource fluorometric screening kit. CETP inhibition decreases fluorescence intensity by reducing lipid transport from donor to acceptor particles. Dimethyl sulfoxide (DMSO) was used to dissolve the synthesized derivatives to prepare 10 mM stock solutions, which were then diluted with water to the required concentrations. The final DMSO concentration was adjusted to 0.1%. Human CETP (5 µL) was mixed with the inhibitor (2 µL) and incubated for 10 minutes. Donor and acceptor particles (5 µL each) were then added, and the volume was adjusted to 200 µL with assay buffer. The final inhibitor concentration in the well was 10 µM. The mixture was incubated at 37 °C for 30 minutes in a covered, sealed plate. The outer wells were avoided to prevent evaporation and temperature gradients. Fluorescence intensity was measured using an FLX800TBI microplate fluorometer (BioTek, USA) at emission and excitation wavelengths of 511 and 480 nm, respectively. Residual CETP activity was determined for each compound by comparing CETP activity in the presence and absence of the test compound. Control samples (blanks) were tested without CETP. Anacetrapib was used as a positive inhibitor. Positive controls were tested to assess the degree of CETP inhibition by 0.1% DMSO. DMSO did not affect CETP. Negative controls lacking human serum CETP were used as background. The signal-to-background ratio was 1.5. All measurements were conducted in duplicate (n = 2), and the mean and standard deviation were calculated using Excel (39, 40). The percentage of CETP inhibition was calculated using the following formula:

$$\%\mathrm{Inhibition}=\left(1-\frac{\mathrm{Inhibitor}\mathrm{read}-\mathrm{Blank}\mathrm{read}}{\mathrm{Positive}\mathrm{control}-\mathrm{Negative}\mathrm{control}}\right)\times 100\%$$

To determine the IC_50_ values of the most active compounds (7f-7j), their activity was tested at concentrations of 10, 1.0, and 0.1 µM. CETP inhibition (%) was then plotted against log concentration. IC_50_ values were calculated from the equation of the dose-response line.

### 3.5. In Silico Study

#### 3.5.1. Preparation of Protein and Ligand Structures

The X-ray crystal structure of CETP (PDB ID: 4EWS) (41) was retrieved from the RCSB Protein Data Bank. The CETP structure was refined and energy-minimized using the Protein Preparation algorithm in Schrödinger Enterprise to improve hydrogen-bonding interactions.

The designed compounds (ligands) were built using the coordinates of the cocrystallized ligand (0RP) in the 4EWS structure (41). Ligands were drawn using the Maestro build script and energy-minimized using the MacroModel tool with the OPLS2005 force field.

#### 3.5.2. Induced-Fit Docking

The cocrystallized ligand 0RP was used as the centroid in the 4EWS binding cleft. The van der Waals radii of both CETP and the ligands were scaled down to 0.5 to allow flexible docking. Other parameters were kept at their default settings. The ligand geometry with the best extra-precision (XP) Glide score was selected. Binding energy was expressed as a docking score (kcal/mol). A more negative docking score indicates a stronger binding interaction.

## 4. Results

### 4.1. Chemistry

Figure 3 shows the preparation of p-methoxybenzene sulfonamides 6a-6j and 7a-7j.

**Figure 3.**
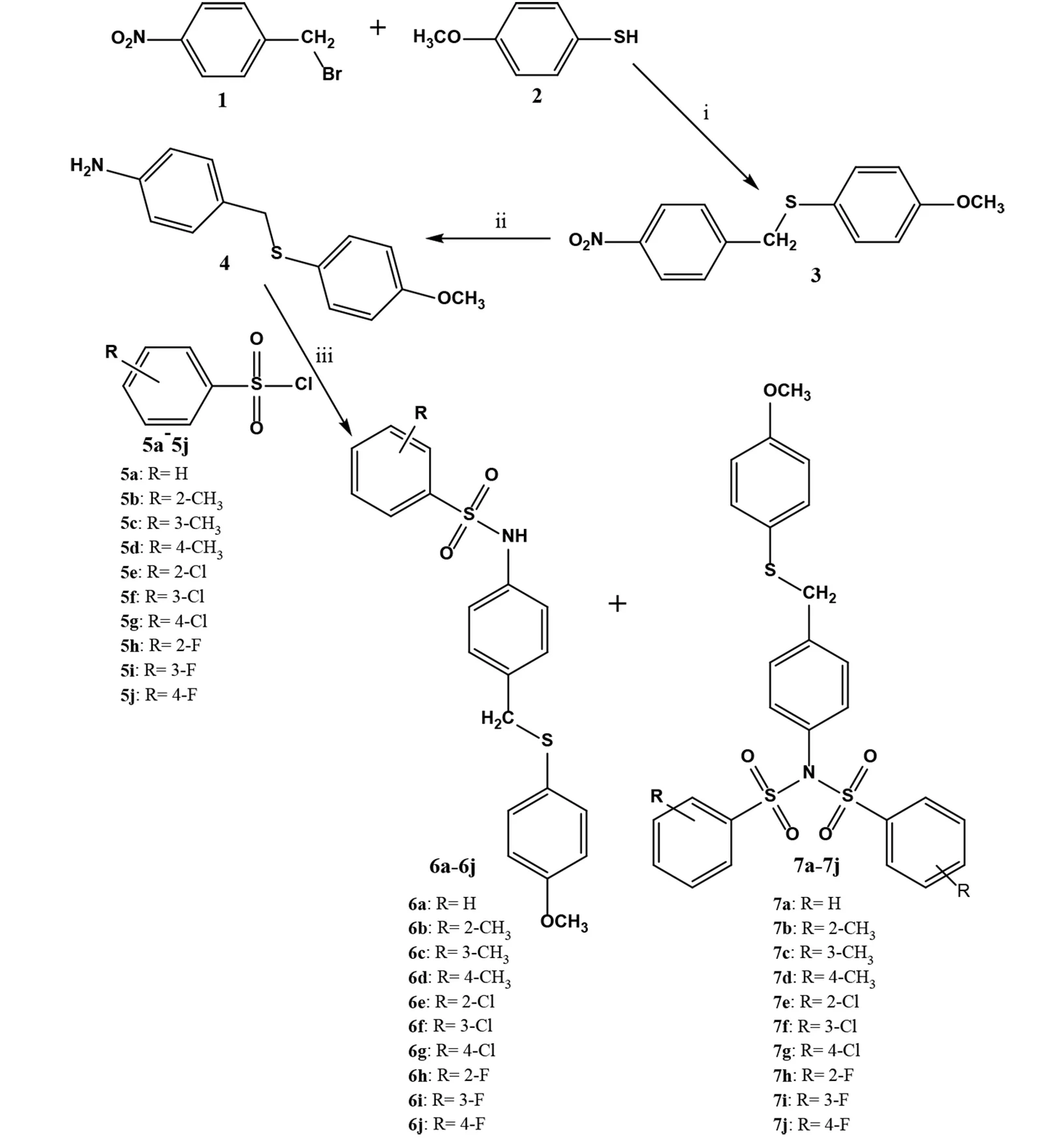
Preparation of the target p-methoxylated benzene sulfonamides 6a-6j and 7a-7j. (i) DCM, TEA, 1 h at room temperature; (ii) acetonitrile, water, NaBH_4_, CoCl_2_·6H_2_O, 30 - 45 min at 60 - 70 °C; and (iii) DCM, TEA, 1 - 3 days at room temperature.

In the synthetic pathway, 4-methoxythiophenol (2) acts as a nucleophile, with its thiol group attacking the partially positive methylene carbon of benzyl bromide 1 to yield phenylsulfane 3. The nitro group of intermediate 3 is reduced to an amine using NaBH_4_ in the presence of CoCl_2_·6H_2_O as a catalyst to yield benzenamine 4. Finally, the amine group of intermediate 4 acts as a nucleophile and attacks the electron-deficient sulfur atom of a sulfonyl chloride (5a-5j), forming 6a-6j. These compounds react with an additional sulfonyl chloride molecule to yield disulfonamides 7a-7j.

Compounds 6 and 7 were synthesized in the same reaction mixture, with compound 6 forming first, followed by compound 7. The reaction was monitored by TLC and continued until the spot intensities of both compounds remained constant. The compounds were then separated by column chromatography. The formation ratio of 6 to 7 ranged from 0.285 for 6c and 7c to 1.224 for 6a and 7a, reflecting the different substitutions on the sulfonamide aromatic ring. Compound 7 was generally favored in 80% of the reactions.

### 4.2. Spectroscopic Characterization of Compounds 4, 6a-6j, and 7a-7j

The ^1^H-NMR data (Table 1) showed that the CH_3_ protons of 6b-6d appeared as a single peak at 2.33 - 2.57 ppm, integrating to 3 H. In 7b-7d, they appeared as a single peak at 2.24 - 2.49 ppm, integrating to 6 H. The OCH_3_ protons appeared as a single peak at 3.73 ppm in intermediate 4, at 3.67 - 3.73 ppm in 6a-6j, and at 3.31 - 3.84 ppm in 7a-7j, integrating to 3 H. The CH_2_ protons appeared as a single peak at 3.94 ppm in 4, at 3.89 - 4.11 ppm in 6a-6j, and at 4.08 - 4.22 ppm in 7a-7j, integrating to 2 H. The NH_2_ protons of 4 appeared as a single peak at 4.99 ppm, integrating to 2 H. The aromatic protons of 4 appeared at 6.46 - 7.26 ppm with coupling constants of 10.0 - 12.0 Hz and an integration of 8 H. The aromatic protons of 6a-6j appeared at 6.77 - 8.18 ppm with different coupling patterns and reported integrations of 12, 13, or 16 H. In 7a-7j, they appeared at 6.84 - 7.98 ppm, integrating to 16 or 18 H. Aromatic coupling constants ranged from 3.45 to 11.0 Hz in 6a-6j and from 3.5 to 14.9 Hz in 7a-7j. Sulfonamide protons appeared as a single peak at 10.14 - 10.65 ppm in 6a-6i, integrating to 1 H.

**Table 1.** ^1^H-NMR Data for the Synthesized Compounds

Type of Protons and Compounds	Chemical Shift (ppm)	Integration
**CH_3_**		
6b-6d	2.33 - 2.57	3 H
7b-7d	2.24 - 2.49	6 H
**OCH_3_**		
4	3.73	3 H
6a-6j	3.67 - 3.73	3 H
7a-7j	3.31 - 3.84	3 H
**CH_2_**		
4	3.94	2 H
6a-6j	3.89 - 4.11	2 H
7a-7j	4.08 - 4.22	2 H
**NH_2_**		
4	4.99	2 H
**Sulfonamide NH**		
6a-6i	10.14 - 10.65	1 H
**Aromatic H**		
4	6.46 - 7.26, J = 10 - 12 Hz	8 H
6a-6j	6.77 - 8.18, J = 3.45 - 11 Hz	12, 13, or 16 H
7a-7j	6.84 - 7.98, J = 3.5 - 14.9 Hz	16 or 18 H

The ^13^C-NMR data (Table 2) showed that the CH_3_ carbon signals of 6b-6d appeared at 20.2 - 21.4 ppm, with reported assignments of 1 carbon for 6b and 6c and 2 carbons for 6d. In 7b-7d, the CH_3_ carbon signals appeared at 20.3 - 21.6 ppm, corresponding to 2 carbons. The CH_2_ carbon signal appeared at 39.4 ppm in 4, at 38.9 - 39.9 ppm in 6a-6j, and at 38.8 - 39.0 ppm in 7a-7j, corresponding to 1 carbon. The OCH_3_ carbon signal appeared at 55.6 ppm in 4, at 55.6 - 55.7 ppm in 6a-6j, and at 55.6 - 56.0 ppm in 7a-7j, corresponding to 1 carbon. Aromatic carbon signals appeared at 114.2 - 159.0 ppm in 4, at 111.1 - 161.1 ppm in 6a-6j, and at 115.1 - 166.9 ppm in 7a-7j.

**Table 2.** ^13^C-NMR Data for the Synthesized Compounds

Type of Carbons and Compounds	Chemical Shift (ppm)	Integration
**CH_3_**		
6b-6c	20.2 - 21.3	1 C
6d	21.4	2 C
7b-7d	20.3 - 21.6	2 C
**CH_2_**		
4	39.4	1 C
6a-6j	38.9 - 39.9	1 C
7a-7j	38.8 - 39.0	1 C
**OCH_3_**		
4	55.6	1 C
6a-6j	55.6 - 55.7	1 C
7a-7j	55.6 - 56.0	1 C
**Aromatic C**		
4	114.2 - 159.0	12 C
6a-6j	111.1 - 161.1	18 C
7a-7j	115.1 - 166.9	24 C

The IR spectra showed the NH_2_ group of 4 as two sharp peaks at 3387 and 3321 cm^-1^. A single sharp NH peak was identified at 3253 - 3317 cm^-1^ in 6a-6j. Aromatic CH peaks were observed at 3014 - 3103 cm^-1^ in 6a-6j and at 3024 - 3167 cm^-1^ in 7a-7j. Aliphatic CH peaks were observed at 2920 - 2962 cm^-1^ in 4, at 2831 - 2966 cm^-1^ in 6a-6j, and at 2833 - 2964 cm^-1^ in 7a-7j. Aromatic C=C peaks were observed at 1437 - 1625 cm^-1^ in 4, at 1442 - 1614 cm^-1^ in 6a-6j, and at 1431 - 1595 cm^-1^ in 7a-7j. Sulfonyl-group peaks were observed at 1176 cm^-1^ in 4, at 1149 - 1168 cm^-1^ in 6a-6j, and at 1157 - 1174 cm^-1^ in 7a-7j.

High-resolution mass spectrometry of the purified samples was also used to confirm the molecular masses and identities of 6a-6j and 7a-7j. The measured masses agreed closely with the theoretically predicted masses.

### 4.3. CETP Inhibitory Activity Assay

As shown in Table 3, the CETP inhibitory activity of compounds 6a-6j and 7a-7j was assessed at 10 µM. In general, the bioassay data showed that the presence of four aromatic rings in the inhibitor structure enhanced CETP inhibitory activity compared with the three-ring analogues: 7a showed 95% inhibition, whereas 6a showed 17% inhibition. This may be attributable to the enhanced lipophilicity of 7a-7j, which is consistent with the hydrophobic nature of the CETP binding pocket (Figure 3 and Table 3).

**Table 3.** CETP Inhibitory Bioactivities of Methoxylated Sulfonamides 6a-6j and 7a-7j ^a^

Compounds	Inhibition at 10 µM (%)	IC_50_ (µM)
**6 ^a^**	17.3 ± 0.8	ND
**6b**	42.0 ± 1.1	ND
**6c**	51.8 ± 2.1	ND
**6d**	39.2 ± 0.9	ND
**6e**	90.6 ± 1.5	ND
**6f**	39.0 ± 1.2	ND
**6g**	89.1 ± 1.9	ND
**6h**	34.2 ± 1.5	ND
**6i**	34.2 ± 1.3	ND
**6j**	0.00 ± 0.0	ND
**7 ^a^**	95.0 ± 0.9	ND
**7b**	54.1 ± 1.3	ND
**7c**	76.7 ± 1.1	ND
**7d**	29.7 ± 1.1	ND
**7e**	50.2 ± 1.6	ND
**7f**	100.0 ± 1.7	0.84
**7g**	100.0 ± 1.1	1.73
**7h**	100.0 ± 1.6	2.46
**7i**	100.0 ± 1.6	6.11
**7j**	100.0 ± 1.2	0.55
**Anacetrapib**	100 (at 0.08 µM) ± 1.3	0.01

^a^ Values are expressed as mean ± SD. n = 2. Abbreviations: CETP, cholesteryl ester transfer protein; ND, not determined.

Among compounds 6a-6j, the chlorinated derivatives 6e (2-Cl) and 6g (4-Cl) showed promising CETP inhibition of approximately 90% at 10 µM. The enhanced activities of these inhibitors may be attributable to the bulky, lipophilic, electron-withdrawing chlorine atom in an orientation favorable for fitting the CETP binding site.

The halogenated derivatives 7f-7j showed the greatest CETP inhibition, reaching 100% at 10 µM; 7j had an IC_50_ of 0.55 µM. Inhibitor 7e (2-Cl) showed less inhibition, which may be attributable to the steric effect of the ortho-chloro group in the binding pocket. The methylated analogues 7b-7d were less active than the halogenated inhibitors 7f-7j, possibly because of the electron-donating nature of the methyl moiety.

### 4.4. Docking Study

To investigate the binding interactions of anacetrapib, the cocrystallized ligand 0RP (torcetrapib), and compounds 6a-6j and 7a-7j with CETP, induced-fit docking (IFD) studies (42, 43) were conducted against the CETP binding domain (PDB ID: 4EWS) (41). The IFD analysis showed that torcetrapib, anacetrapib, 6a-6j, and 7a-7j fit within the CETP binding site (Figure 4).

**Figure 4.**
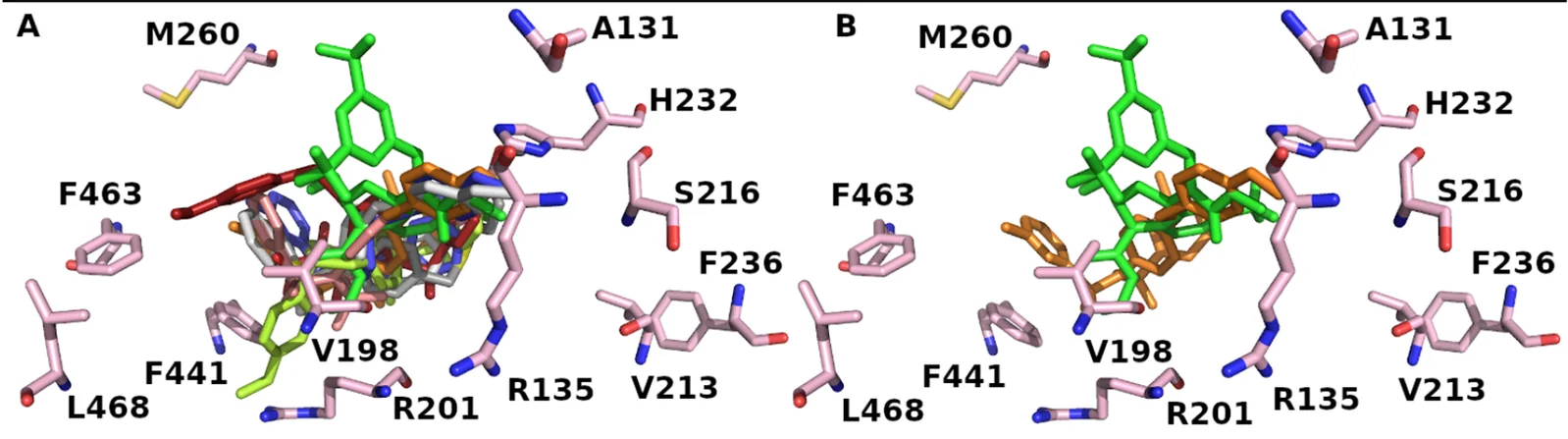
(A) Cholesteryl ester transfer protein (CETP) binding site containing the IFD-generated structures of 7a-7j. (B) Superposition of the IFD-generated geometry of 7g (brown) and the cocrystallized ligand torcetrapib (green). Amino acid atoms are colored as follows: C, pink; O, red; N, blue; and S, yellow. Hydrogen atoms and selected binding residues are omitted for clarity. Image visualized using PyMOL.

The IFD studies showed that hydrophobic interactions guide ligand/4EWS complex formation (Figure 5). The sulfonamides form hydrogen bonds with H232 and R201 (Table 4). Previous docking studies have highlighted the importance of R201 and H232 in ligand/CETP binding interactions (37, 38, 40, 44-48). Higher binding scores were observed for analogues 7a-7j than for 6a-6j, highlighting the importance of incorporating four aromatic rings to promote inhibitory activity.

**Table 4.** Induced-fit docking (IFD) Scores (kcal/mol) and Hydrogen Bonds ^a^

Compounds	Docking Score (kcal/mol)	Hydrogen-Bond Residue
**6 ^a^**	-8.47	NA
**6b**	-9.00	H232
**6c**	-10.17	NA
**6d**	-9.20	R201
**6e**	-9.04	R201
**6f**	-8.76	R201
**6g**	-8.74	R201
**6h**	-8.96	NA
**6i**	-9.44	NA
**6j**	-8.82	R201
**7 ^a^**	-9.63	R201
**7b**	-9.74	NA
**7c**	-10.06	R201
**7d**	-9.90	R201
**7e**	-10.19	R201
**7f**	-10.35	R201
**7g**	-10.14	NA
**7h**	-9.69	NA
**7i**	-9.38	NA
**7j**	-10.01	R201
**Torcetrapib (0RP)**	-9.52	R201
**Anacetrapib**	-11.52	NA

^a^ 6 and 7 refer to compounds 6a and 7a, respectively. Abbreviation: NA, not available.

**Figure 5.**
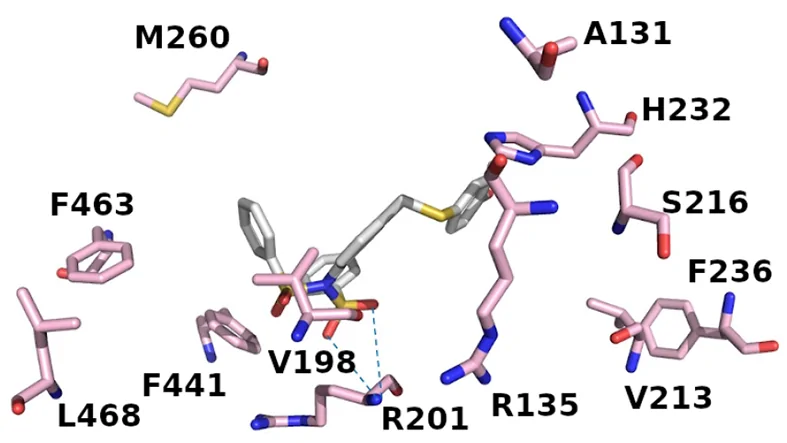
IFD-generated poses of 7a in the cholesteryl ester transfer protein (CETP) binding site. The hydrogen bond is shown as a blue dotted line. Selected binding residues are shown, and hydrogen atoms are omitted for clarity. Image prepared using PyMOL.

To evaluate the performance of the IFD program, the docked pose of torcetrapib in CETP (PDB ID: 4EWS) (41) was compared with its cocrystallized structure. Figure 6 shows the superposition of the IFD-generated torcetrapib pose and its original coordinates in CETP. The root mean square deviation of the torcetrapib heavy atoms between the IFD-generated and original poses was 0.9522 Å. This result indicates that IFD can reproduce the native pose in the crystal structure and identify the ligand-binding conformation.

**Figure 6.**
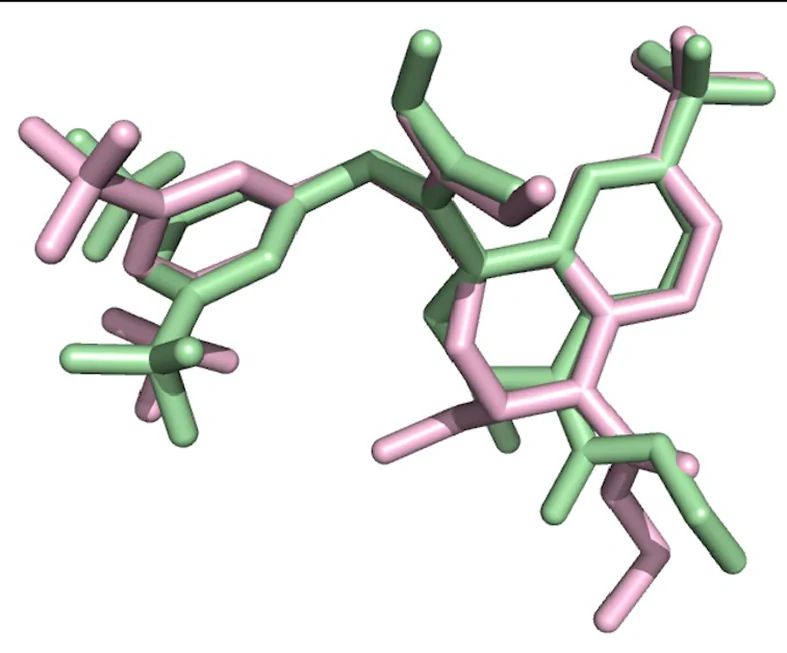
Superposition of the original cocrystallized torcetrapib ligand coordinates (green) and its IFD-generated geometry (purple) in cholesteryl ester transfer protein (CETP). Image prepared using PyMOL.

The biological data showed that compounds 7f, 7g, 7h, 7i, and 7j had the greatest CETP inhibitory activity, highlighting the importance of incorporating a fourth aromatic ring substituted with m-Cl (7f), p-Cl (7g), o-F (7h), m-F (7i), or p-F (7j). These results indicate that electronegativity is crucial for promoting activity. The activity of the o-Cl derivative 6e suggests that steric features impede the proper positioning of the o-Cl derivative 7e and support the importance of four aromatic rings. The biological data support the higher binding scores of 7a-7j and suggest that the substitution position affects accommodation within the binding domain.

### 4.5. Pharmacophore Mapping

The structural features of the synthesized sulfonamides were evaluated by mapping them against an adopted pharmacophore model for CETP inhibitors (37). The derivatives matched the fingerprint of active CETP inhibitors (Figure 7), helping to explain their docking scores against the CETP binding domain. Their positioning within the CETP binding domain supports their potential CETP inhibitory activity.

**Figure 7.**
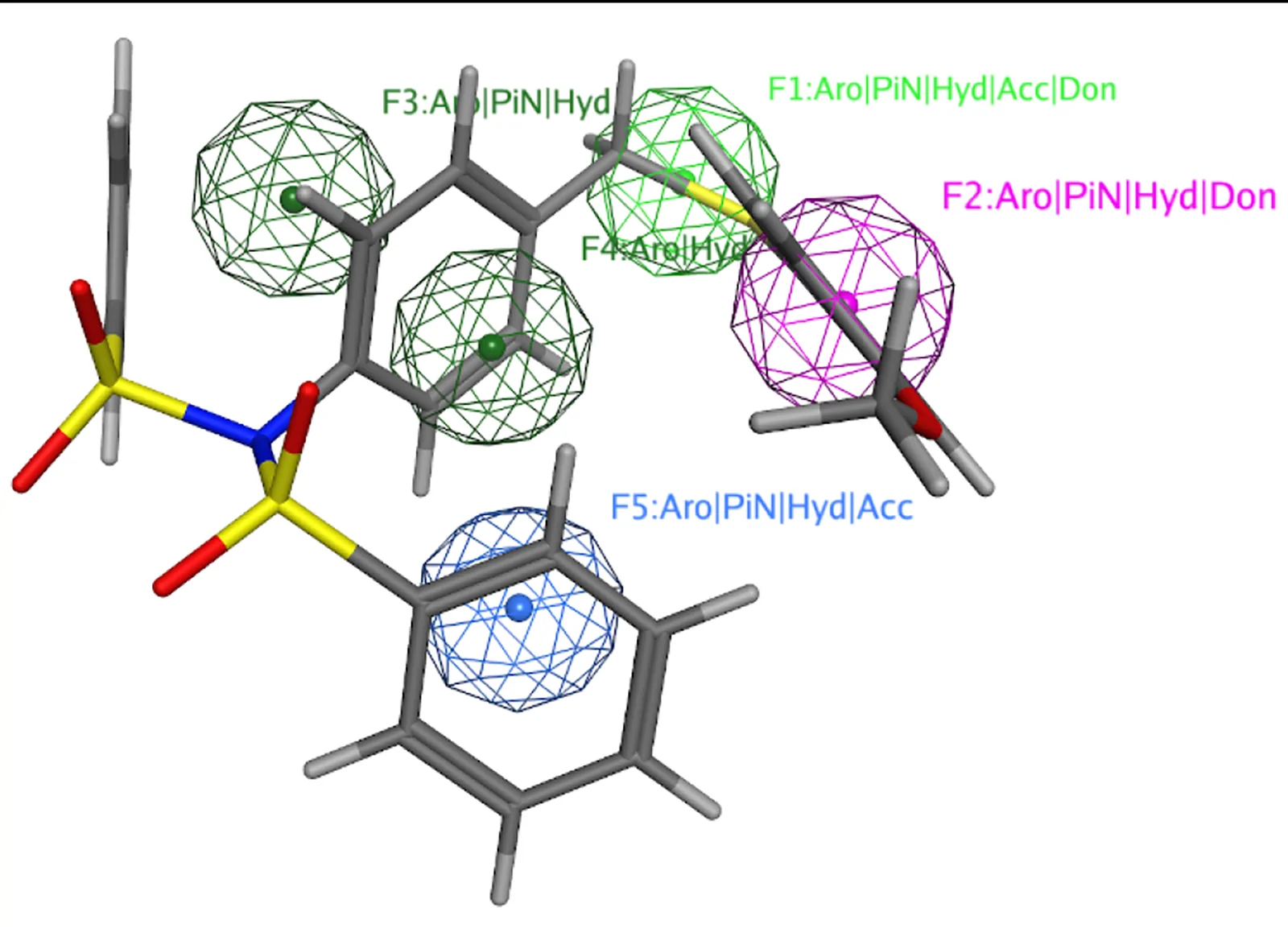
Mapping of 7a to the cholesteryl ester transfer protein (CETP) inhibitor pharmacophore model. Aro, aromatic ring; Acc, hydrogen-bond acceptor; Don, hydrogen-bond donor; Cat, cationic group; PiN, π-ring; and Hyd, hydrophobic group. Image visualized using Molecular Operating Environment.

## 5. Discussion

This study investigated the CETP-inhibitory activities of a new series of methoxylated diaryl sulfonamides. The synthetic pathway involved nucleophilic addition-elimination reactions and a reduction step. The synthesized compounds were fully characterized and structurally confirmed using IR, NMR, and HR-MS. In recent years, CETP has been investigated as an important target for treating dyslipidemia in patients at cardiovascular risk. Researchers have also focused on compounds that target CETP to improve lipid profiles and potentially reduce cardiovascular risk. Anacetrapib demonstrated cardiovascular benefits and favorable lipid-modifying effects (49). Newer CETP inhibitors, such as obicetrapib, have shown encouraging lipid improvements, reducing LDL cholesterol levels by nearly 30% (50).

Our results also showed greater CETP inhibition and higher binding scores for analogues 7a-7j than for 6a-6j, highlighting the effect of incorporating four aromatic rings on inhibitory activity. The target compounds also matched the pharmacophore fingerprint of active CETP inhibitors.

This study has several limitations. Molecular docking and pharmacophore analyses provide predictive insights into ligand-target interactions but do not fully account for protein flexibility or dynamic biological environments (51-53). Experimental validation was limited to in vitro assays using technical replicates, which may not fully reflect in vivo conditions. The number of compounds evaluated was also limited, restricting comprehensive structure-activity relationship analysis. Further studies using expanded compound libraries, target-validation experiments with biological replicates, and in vivo models are warranted to confirm and extend these findings.

### 5.1. Conclusions

A new series of 4-methoxy-substituted benzene sulfonamides (6a-6j and 7a-7j) was successfully synthesized and structurally characterized as potential CETP inhibitors. Compounds 7f-7j showed the greatest inhibitory activity, achieving 100% inhibition at 10 µM; 7j had an IC_50_ of 0.55 µM. Biological evaluation showed that introducing a fourth aromatic ring markedly improved activity, likely because of the hydrophobic nature of CETP. Higher IFD binding scores were also observed for analogues 7a-7j than for their 6a-6j counterparts, highlighting the importance of incorporating four aromatic rings in promoting inhibitory activity. Pharmacophore mapping further confirmed that the structural features of the synthesized analogues aligned with the recognized pharmacophore model for active CETP inhibitors, highlighting their potential for further development.

## Data Availability

The dataset presented in the study is available on request from the corresponding author during submission or after publication.
